# Biodegradation of Synthetic Polymers Used in Consolidation of Deteriorated Limestone Monuments

**DOI:** 10.3390/polym18101218

**Published:** 2026-05-16

**Authors:** Shimaa Ibrahim, Moez A. Ibrahim, Dina M. Atwa, Rageh K. Hussein, Hesham Abdulla

**Affiliations:** 1 Museum Sector, Ministry of Antiquities, Cairo P.O. Box 12561, Egypt; shimaa_sa3d53@yahoo.com; 2 Physics Department, College of Science, Imam Mohammad Ibn Saud Islamic University (IMSIU), Riyadh 11623, Saudi Arabia; maimohammed@imamu.edu.sa (M.A.I.); rahussein@imamu.edu.sa (R.K.H.); 3 Department of Laser Interaction with Matters, Laser Institute for Research and Applications, Beni-Suef University, Beni-Suef P.O. Box 62517, Egypt; 4 Department of Botany and Microbiology, Faculty of Science, Suez Canal University, Ismailia P.O. Box 41522, Egypt; hesham_abdulla@science.suez.edu.eg

**Keywords:** acrylic resins, limestone conservation, microbial biodegradation, Paraloid, biodeterioration, stone cultural heritage

## Abstract

Synthetic polymers are widely used in stone conservation, yet their long-term biological stability remains insufficiently evaluated. This study investigates the microbial susceptibility of three commonly used acrylic consolidants, Paraloid B-72, B-66, and B-44, applied to deteriorated limestone. Bacteria, fungi, and actinomycetes were isolated from a deteriorated limestone false door and screened for acid production. From each microbial group, only the strong acid-producing isolates were selected for further investigation, including evaluation of their ability to utilize the three Paraloid resins as sole carbon sources and their deterioration potential on limestone cubes before and after consolidation. Deterioration was assessed by weight loss, compressive strength testing, stereomicroscopy, scanning electron microscopy (SEM), X-ray diffraction (XRD), and Fourier transform infrared spectroscopy (FTIR). All selected strong acid-producing isolates demonstrated the ability to grow on the tested polymers, confirming their biodegradation potential. Mixed microbial cultures caused greater weight loss and compressive strength reduction than single isolates, attributed to synergistic metabolic interactions. Among the consolidants, Paraloid B-72 showed the highest susceptibility to microbial attack, while Paraloid B-66 exhibited comparatively greater resistance, attributed to the steric hindrance of its isobutyl side groups and higher surface hydrophobicity. FTIR and XRD analyses confirmed ester bond hydrolysis, progressive gypsum formation, and structural alteration of the limestone substrate. These findings demonstrate that acrylic consolidants commonly used in stone conservation are not biologically inert and may actively contribute to biodeterioration under microbial colonization, highlighting the need for developing bio-resistant conservation materials.

## 1. Introduction

Cultural heritage materials represent an essential record of past human societies [1]. These tangible remains provide valuable evidence of daily life, social structures, artistic traditions, and technological developments in manufacturing and craftsmanship [1,2]. By studying the materials used and the techniques applied in their production, researchers can better understand how earlier communities interacted with their environment and developed their cultural identities [1,2,3].

However, cultural heritage objects are highly vulnerable to deterioration over time. Environmental factors such as fluctuations in temperature and humidity, exposure to light, air pollution, and physical stress can gradually alter the chemical and physical properties of these materials [4,5]. As a result, different forms of deterioration may occur, including cracking, corrosion, discoloration, structural weakening, and material loss [4,5,6].

In addition to environmental impacts, biological agents also play a significant role in the degradation of heritage materials. Microorganisms such as bacteria, fungi, and algae can colonize the surfaces of artifacts and monuments, leading to biodeterioration through staining, enzymatic activity, and the production of acidic metabolic by-products [6,7,8]. These biological processes may accelerate the breakdown of organic and inorganic materials alike. Therefore, understanding the deterioration mechanisms affecting cultural heritage is crucial for developing effective conservation and preservation strategies to protect these valuable records of human history for future generations [6,7,8].

Stone monuments and artifacts are among the most widespread forms of cultural heritage worldwide, and like other types of archeological materials, they are exposed to various forms of deterioration over time, making their conservation and treatment essential for long-term preservation [8]. Although consolidating materials are often applied to strengthen and stabilize deteriorated stone, in many cases these materials may themselves contribute to or accelerate further deterioration [9,10,11,12,13,14]. Synthetic polymers are widely used in the conservation of stone cultural heritage as adhesives, binders, consolidants, and protective coatings due to their ability to improve mechanical strength, enhance cohesion between deteriorated particles, and provide temporary stabilization for fragile stone structures [12,15]. However, despite their practical advantages, these materials may also introduce additional conservation challenges. Many synthetic polymers are organic in nature and can act as a potential carbon source that supports the growth of biological organisms such as bacteria, fungi, algae, and lichens [12,13,14,15,16,17]. The accumulation of these microorganisms on treated stone surfaces may lead to biodeterioration processes, including discoloration, biofilm formation, and the production of acidic metabolic by-products that accelerate the chemical and physical degradation of the stone substrate [18,19]. Consequently, while polymeric consolidants play an important role in stone conservation, their long-term interaction with biological agents must be carefully considered when selecting appropriate treatment materials [19].

Consolidation is defined as the impregnation of deteriorated stone with suitable polymeric materials capable of penetrating the damaged zones and reaching the underlying sound layers, thereby restoring cohesion and enhancing the mechanical integrity of the stone structure [13,20].

Polymeric consolidants and protective coatings are therefore frequently applied to preserve stone artifacts and monuments from further deterioration and environmental weathering [20,21]. An effective protective coating should be compatible with the stone substrate, water-repellent, and able to limit the penetration of harmful chemical agents, while ideally also reducing microbial colonization on treated surfaces [21]. For this reason, monitoring the long-term performance of conservation resins has always been a critical concern for the conservation community, as the durability and stability of treated heritage objects largely depend on the service life of the applied material [19,20,21]. However, increasing evidence suggests that many synthetic polymers may act as potential substrates for heterotrophic microorganisms, including bacteria and fungi [12,13,14,15,16,17]. The susceptibility of polymers to biodegradation is influenced by several factors such as molecular weight, crystallinity, and physical structure [22,23,24,25]. Microbial degradation mechanisms may involve the utilization of the polymer itself or its additives as sources of carbon and energy for microbial metabolism [12,13]. As a result, biodeterioration processes may occur, including the formation of biological films that obscure surface characteristics, the production of organic acids, and enzymatic depolymerization of polymer chains, ultimately affecting both the polymeric material and the treated stone substrate [12,13,14,15,16,17].

Such acrylic resins, particularly those belonging to the Paraloid family, are among the most commonly used materials in stone conservation because of their transparency, stability, and relative reversibility [26,27]. Early research by Nugari, M.P. and Priori, G.F. (1985) [28] evaluated the resistance of the acrylic resin Paraloid B-72 to microbial attack using agar plate count tests and suggested that such polymers were relatively resistant to biodeterioration [26,28].

Similarly, Wenjuan Li et al. (2021) investigated hybrid conservation coatings composed of Paraloid B-72 modified with TiO_2_ nanoparticles applied to sandstone monument [29]. Although the incorporation of nanoparticles improved certain protective properties, the study also highlighted those environmental factors such as UV radiation may accelerate degradation of the polymer matrix, potentially influencing its long-term interaction with microorganisms [29].

In another study, Mohamed Moustafa Ibrahim et al. (2021) examined acrylic nanocomposite consolidants based on Paraloid B-72 modified with TiO_2_ and ZnO nanoparticles for consolidation of pottery samples [30]. Their results showed that the addition of nanoparticles could improve durability and introduce antimicrobial effects, suggesting that modifications of traditional polymers may enhance their resistance to microbial colonization [30]. Later studies began to challenge this assumption. For instance, Ioana Gomoiu et al. (2022) demonstrated that several microorganisms, including melanized fungi, are capable of colonizing surfaces treated with acrylic consolidants such as Paraloid B-72 [31]. Their findings indicated that some polymeric coatings may support microbial growth and biofilm formation, particularly under conditions of high humidity and limited vapor permeability [31].

**Figure 1:**
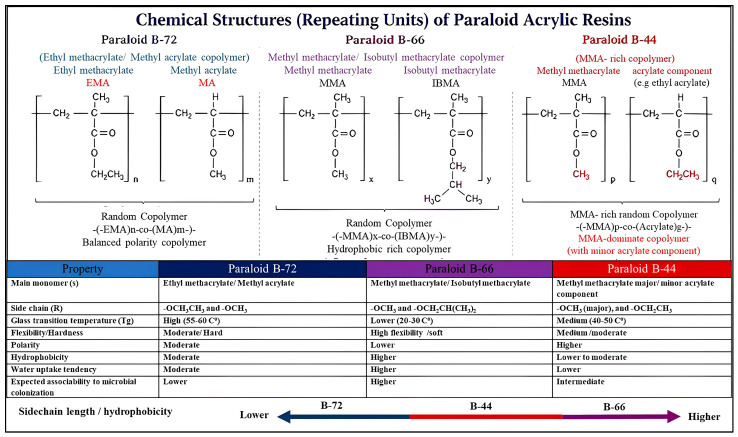
Repeating unit structures and selected properties of Paraloid B-72, B-66, and B-44. Variations in copolymer composition and side-chain structure govern polarity, hydrophobicity, and thermal behavior, which in turn influence microbial adhesion and biodeterioration susceptibility.

Despite the growing number of studies examining the durability and environmental performance of polymer consolidants, relatively limited attention has been given to the direct interaction between microorganisms naturally present on archeological stone and commonly used acrylic polymers. Most previous research has focused primarily on the physical and chemical aging of these materials, while fewer studies have evaluated their potential to serve as carbon sources for heterotrophic microorganisms or assessed the resulting impact on the integrity of polymer-treated stone substrates. Consequently, the microbial susceptibility of widely used acrylic resins—particularly Paraloid B-72, Paraloid B-66, and Paraloid B-44—when applied to archeological limestone remains insufficiently investigated. The three Paraloid acrylic resins examined in this study differ substantially in their chemical composition and physicochemical properties. Paraloid B-72 is a copolymer of ethyl methacrylate and methyl acrylate (70:30 ratio) characterized by relatively high glass transition temperature (Tg) and moderate polarity, which contributes to its stability and resistance to environmental degradation. It widely used in stone conservation due to its chemical stability and reversibility [26,27]. Paraloid B-44 is a copolymer of methyl methacrylate and ethyl acrylate [27,32,33,34]; its different monomer ratio results in the comparatively higher accessibility of ester groups within the polymer backbone, rendering it more vulnerable to enzymatic hydrolysis by microbial esterases [35,36,37]. Paraloid B-66 is a copolymer of methyl methacrylate and isobutyl methacrylate [27,33,34]; its bulky isobutyl side groups create significant steric hindrance around the ester bond and confer higher surface hydrophobicity [22,23,24,25], together limiting microbial surface colonization and enzymatic accessibility. These structural differences form the basis for interpreting the differential biodegradation patterns observed throughout this study. Figure 1 illustrates the comparison between the three mentioned Paraloids, clarifying their different chemical structures, physical and thermal properties.

Therefore, the present study aims to not only address this gap by investigating the microbial susceptibility of three commonly used acrylic polymers (Paraloid B-72, B-66, and B-44), but also to provide a clearer understanding of their role in biodeterioration processes affecting limestone. Unlike previous studies, this work presents (i) a direct comparative evaluation of three Paraloid resins applied to the same limestone substrate under controlled conditions, (ii) an assessment of both individual microbial isolates and mixed microbial consortia to better simulate natural colonization and synergistic effects, and (iii) an integrated analytical approach combining SEM, XRD, FTIR, and mechanical testing to directly link polymer biodegradation with the structural and mineralogical deterioration of the stone. In particular, this study provides quantitative XRD evidence of enhanced gypsum formation following microbial activity on polymer-treated limestone, highlighting that such consolidants may contribute to deterioration under biological conditions rather than solely acting as protective materials.

## 2. Materials and Methods

### 2.1. Studying Monument and Sampling Procedures

**Figure 2:**
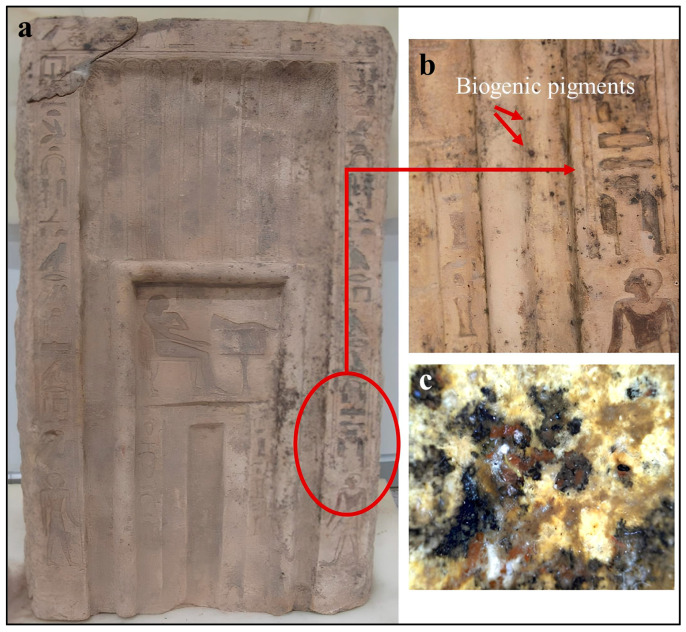
Limestone false door used as the source of microbiological sampling. (**a**) General view of the deteriorated limestone false door showing surface weathering and areas of discoloration. (**b**) Magnified view of the swapped decorated surface illustrating dark spots attributed to biogenic pigments produced by microbial colonization. (**c**) Stereomicroscopic observation of the deteriorated surface revealing heterogeneous deposits, microbial growth, and mineral accumulations associated with biodeterioration processes.

Microbiological samples were collected from a deteriorated limestone false door currently housed at the Grand Egyptian Museum. The artifact measures approximately 51 cm in length, 12 cm in width, and 96 cm in height. Visual examination revealed several deterioration features affecting the stone surface, including dark discoloration, bio-pitting, micro-cracks, and localized accumulations of salt crystals (shown in Figure 2a). In addition, small dark spots consistent with biogenic pigmentation were observed on different areas of the decorated surface, suggesting possible microbial colonization (Figure 2b). The ambient temperature inside the Conservation Center of the Grand Egyptian Museum at the time of sampling ranged between 23 and 25 °C, and relative humidity ranged between 50 and 55%, as recorded by a calibrated digital hygrometer. These controlled indoor conditions are consistent with the standard preservation environment maintained by the museum.

To investigate the associated microbial communities, samples were collected from the outer friable and deteriorated zones of the limestone surface. Sterile cotton swabs were primarily used to obtain microbiological samples, while additional micro-samples were carefully taken using a sterile scalpel and adhesive tape in order to recover surface deposits and loosely attached microbial growth. Sampling targeted areas showing visible biodeterioration symptoms to maximize the recovery of active microorganisms associated with the stone surface.

### 2.2. Isolation and Enumeration of Microorganisms

The collected samples obtained using different sampling techniques were processed as follows. Adhesive tape strips were gently pressed onto the deteriorated stone surface to capture surface-associated microorganisms. The tape strips were then carefully removed and immediately placed onto the surface of sterile agar plates before incubation [38,39,40]. Swab samples were transferred into sterile phosphate buffer supplemented with 0.001% Tween 80 to facilitate the detachment of microbial cells from the swab fibers. The suspension was vigorously mixed for 30 min using a vortex mixer (VM-1000) to ensure adequate dispersion of the microorganisms [41]. For stone fragments, the collected samples were aseptically crushed into fine powder using a sterile mortar. The powdered material was then suspended in sterile phosphate buffer containing 0.001% Tween 80 and agitated for 30 min using an electrical shaker to release the associated microorganisms into the suspension. Microbial enumeration was performed using the spread plate technique, where 0.1 mL of the prepared suspension was spread onto the surface of the appropriate culture media plates [42].

Different selective media were used for the isolation and enumeration of microbial groups. Nutrient agar (NA) was used for bacterial isolation according to MacFaddin (1985) [39]. Starch casein agar (SC) supplemented with cycloheximide was used for the isolation of actinomycetes following Küster and Williams (1964) [40]. Czapek–Dox agar was used for the isolation of fungi [41]. After inoculation, the plates were incubated at 28 °C. Bacterial colonies were counted after 1–2 days, fungal colonies after 5–7 days, while actinomycete colonies were recorded after 14 days of incubation.

### 2.3. Screening for Acids Production

To evaluate the potential role of the recovered microorganisms in stone biodeterioration, the obtained isolates were screened for their ability to produce organic acids during growth. Bacterial isolates were cultured on nutrient agar medium supplemented with 1.0% glucose and 0.007% bromocresol purple as a pH indicator. A change in the color of the medium from purple to yellow indicated acid production [42,43]. While actinomycete and fungal isolates were examined using a basal mineral medium containing (NH_4_)_2_HPO_4_ (1 g), KCl (0.2 g), MgSO_4_·7H_2_O (0.2 g), bromocresol purple (0.07 g), and agar (18 g) dissolved in 1000 mL of distilled water. The medium was supplemented with 1.0% (*w*/*v*) glucose as the carbon source [41].

Acid production was indicated by a color change in the medium from purple to yellow or by the formation of a yellow halo surrounding the colonies due to a decrease in pH caused by acidic metabolites [43].

### 2.4. Utilization of Paraloids as Sole Carbon Sources

The ability of the recovered bacterial, actinomycete, and fungal isolates to utilize these polymers (Paraloid B-72, Paraloid B-66, and Paraloid B-44) as sole carbon sources was examined using carbon-utilization media [44]. Bacterial and actinomycete isolates were cultured on ISP9 carbon utilization agar, whereas fungal isolates were tested on Czapek–Dox agar medium without sucrose [45]. The composition of ISP9 medium was as follows: (NH_4_)_2_SO_4_ (2.64 g), KH_2_PO_4_ (2.38 g), K_2_HPO_4_·3H_2_O (5.65 g), MgSO_4_·7H_2_O (1.0 g), trace salts solution (1.0 mL), agar (15 g), and distilled water (1000 mL). The pH of the medium was adjusted to 6.8–7.0. The trace salts solution consisted of CuSO_4_·5H_2_O (0.64 g), FeSO_4_·7H_2_O (0.11 g), MnCl_2_·4H_2_O (0.79 g), and ZnSO_4_·7H_2_O (0.15 g) dissolved in 100 mL distilled water [44].

The tested polymers were added to the media at a concentration of 1.0% (*w*/*v*) as the sole carbon source according to Shirling and Gottlieb [45]. In parallel, positive controls containing glucose (1.0% *w*/*v*) and negative controls without any carbon source were prepared to ensure the validity of the experiment. In addition, sterile polymer controls were prepared by adding each Paraloid resin at 1.0% (*w*/*v*) to the appropriate media, autoclaving, and incubating under identical conditions without microbial inoculation. No turbidity, colony formation, or detectable mass change was recorded in any of the sterile polymer controls throughout the 21-day incubation period, confirming that the Paraloid resins do not undergo spontaneous abiotic dissolution or hydrolysis under the experimental conditions used.

All inoculated plates were incubated at 28 °C, and microbial growth was monitored after 2, 7, 14, and 21 days of incubation. The appearance of visible growth on polymer-containing media indicated the ability of the tested isolates to utilize the polymers as carbon sources and suggested their potential role in the biodegradation of these conservation materials [45,46,47].

### 2.5. Characterization of Isolates

Two bacterial and two actinomycete isolates, showed abilities to utilize Paraloid as sole carbon source, were characterized by 16S rDNA sequence analysis; this technique was performed in Biotechnology Research Center (Suez Canal University—Egypt). Total genomic DNA of the isolates was extracted according to a slightly modified method of Pitcher et al. (1989) [48]. 16S rDNA was amplified with universal primers and sequenced as previously described by Heyrman and Swings (2001) [49]. On the other hand, the two most active fungal isolates were identified according to the cultural features of the mycelia (e.g., diameter and color) and microscopic observation of their morphological characteristic and structural peculiarities (e.g., spore-bearing structures) according to Gravesen et al. (1994) [50]. using light microscope (Carl Zeiss Microscope, Imager. M1) and compared with the typical criteria present in standard references.

### 2.6. Collection of Stone Specimens for Model Study

The stone specimens used in the following experiments were collected from dumped limestone blocks of an archeological site near an old mosque at EL-Moez Street in Old Cairo-Egypt; stone blocks had been cut into cubes of dimensions 3 cm × 3 cm × 3 cm.

### 2.7. Mixed and Single Inoculation Before Consolidation

Prior to inoculation, limestone cubes were dried overnight at 105 °C, weighed, and sterilized at 150 °C for 3 h to eliminate pre-existing microorganisms. The sterilized cubes were aseptically transferred into sterile glass jars containing 100 mL of Oxoid water peptone medium (0.5%) supplemented with 1% glucose (pH 7.2). For single inoculation, each jar received 10 mL of a bacterial suspension (10^4^ CFU mL^−1^) of the selected isolates, while mixed inoculation was performed by combining equal volumes of bacterial suspensions to produce a mixed microbial inoculum. Similar procedures were applied for actinomycetes and fungi using starch casein broth and Czapek–Dox broth, respectively [51].

All inoculated cubes, together with uninoculated controls, were incubated statically at 28 °C for four weeks to allow microbial colonization and potential biodeterioration of the limestone substrate. After incubation, the cubes were removed, dried, and reweighed to assess weight changes. Any visible surface alterations were documented, and mechanical cleaning was performed using soft brushes and scalpels to remove loosely attached microbial growth and deposits following standard stone conservation protocols [52].

### 2.8. Mixed and Single Inoculation After Consolidation

The synthetic polymers (Paraloid B-72, B-66, and B-44) were prepared at 2% (*w*/*v*) in toluene, consistent with standard conservation practice for stone impregnation treatments. Before consolidation, limestone cubes recovered from the first inoculation step (Section 2.7) were mechanically cleaned using soft brushes and sterile scalpels, dried overnight at 105 °C, and reweighed. The consolidant solution was applied to each cube surface by brush in a single coat until visible surface saturation was achieved. The treated cubes were then left to dry at room temperature for 24 h before reweighing to determine polymer uptake. Three replicate cubes (n = 3) were prepared for each treatment group. After consolidation, all stone cubes were oven-sterilized at 150 °C for 3 h to eliminate any surviving microorganisms prior to the second inoculation step [53].

### 2.9. Evaluation of Polymer Biodegradation and Stone Deterioration

A combination of diagnostic techniques was employed to evaluate the biodegradation of the tested synthetic polymers and the resulting deterioration of the limestone cubes. These techniques were selected to detect both morphological and chemical changes occurring in the polymer-treated stone after microbial colonization.

Surface alterations and microbial colonization were first examined using stereomicroscopy (Discovery V20, Carl Zeiss, Oberkochen, Germany) Images were captured at different magnifications using Axiovision software (AxioVision Rel. 4.8) to document visible changes such as discoloration, surface roughness, biofilm formation, and the development of microcracks [54].

Further investigation of surface morphology was conducted using scanning electron microscopy (SEM) (FEI Quanta 3D 200i, FEI Company, Eindhoven, The Netherlands). SEM analysis allowed detailed observation of microbial colonization, biofilm structures, and microstructural changes in the polymer-treated limestone surface, including pore modifications and degradation features resulting from microbial activity [4,55].

X-ray diffraction (XRD) analysis was carried out to identify possible mineralogical changes in the limestone caused by microbial weathering or polymer degradation. Powdered limestone samples were analyzed using a (PANalytical X’Pert Pro (3040/60) diffractometer, PANalytical, Almelo, The Netherland). Phase identification was performed using X’Pert HighScore software, Plus version 2.2. This analysis helped detect any alteration in mineral phases associated with biodeterioration processes [56].

In addition, Fourier Transform Infrared Spectroscopy (FTIR) was used to investigate chemical changes in the polymeric consolidants after microbial exposure. FTIR analysis enables the identification of functional groups and can reveal structural modifications in the polymer chains, such as oxidation, hydrolysis, or depolymerization resulting from microbial activity [57].

To evaluate the mechanical impact of microbial deterioration, compressive strength tests were performed on the limestone cubes before and after consolidation and microbial inoculation. The tests were conducted using a Shimadzu TRAPEZIUM AG-X, (Shimadzu Corporation, Kyoto, Japan) testing system. The compressive strength (fc) of each specimen was calculated at the maximum load (Fmax), and the relationship between maximum force and displacement was analyzed using TRAPEZIUM-X software (TRAPEZIUM software for AG-X). This test provided quantitative data on the influence of microbial activity and polymer biodegradation on the structural integrity of the treated limestone [58].

## 3. Results

### 3.1. Isolation and Enumeration of Microorganisms

**Figure 3:**
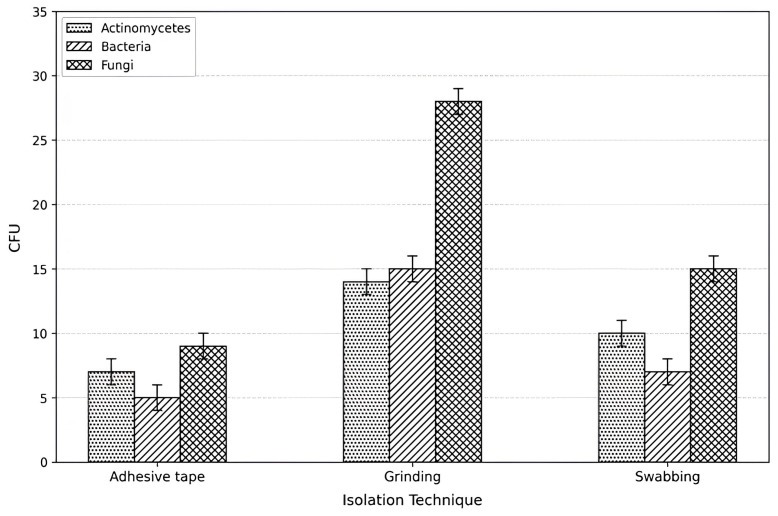
Comparison of microbial counts obtained using different isolation techniques from the limestone false door surface. The grinding method yielded the highest number of recovered microorganisms, followed by swabbing and adhesive tape techniques. In all sampling methods, fungal counts were higher than those of bacteria and actinomycetes. Values are expressed as mean ± SD (n = 3). CFU g^−1^ for the grinding method; CFU cm^−2^ for the adhesive tape and swabbing methods. The small horizontal lines on top of the bars are part of the error bars and indicate the limit of variation or uncertainty in the measured data.

The efficiency of the three sampling techniques used for the isolation of microorganisms from the limestone false door was compared, and the results are presented in Figure 3. The highest microbial counts were obtained using the stone grinding method, which yielded mean counts of 14, 15, and 25 CFU g^−1^ for actinomycetes, bacteria, and fungi, respectively. In contrast, lower counts were recorded using the swabbing and adhesive tape techniques.

The higher microbial recovery obtained from the grinding method can be attributed to its ability to release microorganisms not only from the stone surface but also from the internal pores and microfractures of the limestone matrix. On the other hand, swabbing and adhesive tape methods primarily recover microorganisms present on the outer surface.

In all sampling techniques, fungal counts were consistently higher than those of bacteria and actinomycetes, indicating the predominance of fungal communities on the deteriorated limestone surface.

#### 3.1.1. Acid Production and Utilization of Paraloids

**Figure 4:**
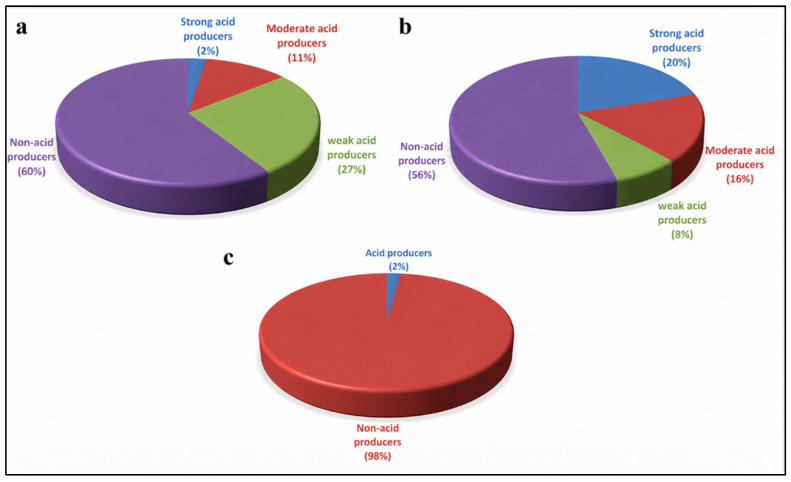
Distribution of acid-producing isolates recovered from the deteriorated limestone false door. (**a**) Acid production pattern among the recovered bacterial isolates showing weak, moderate, and strong acid producers as well as non-acid-producing isolates. (**b**) Acid production capacity of fungal isolates, demonstrating a higher proportion of strong and moderate acid producers compared with bacteria. (**c**) Acid production among actinomycete isolates, where only a very small percentage exhibited acid production while the majority were non-acid producers.

The recovered eubacterial isolates from the limestone false door were screened for their ability to produce acids. The results revealed that 41.6% of the total isolates (102 out of 245) were capable of acid production. The acid-producing isolates showed different levels of activity; 27% were weak producers, 11% were moderate producers, and 2% exhibited strong acid production (Figure 4a).

The fungal isolates were also examined for their acid production potential. Approximately 44.8% of the total isolates (41 out of 145) were able to produce acids. Among them, 8% were weak acid producers, 16% were moderate producers, and 20% were classified as strong acid producers (Figure 4b).

The actinomycete isolates recovered from the limestone false door showed very limited acid production ability. Only 2% of the total isolates were capable of producing acids, while the remaining isolates showed no detectable acid production (Figure 4c).

As illustrated in Figure 3, fungal isolates showed the highest proportion of strong acid producers compared with bacterial and actinomycete isolates. Since no strong acid-producing actinomycete isolates were obtained from the studied artifact, five previously isolated strong acid-producing actinomycetes obtained from deteriorated limestone tombs at Tell Basta, Zagazig City were included in the present study to evaluate their biodeterioration potential. These isolates were *Streptomyces exfoliatus*, *Nocardiopsis alborubida*, *Nocardioides luteus*, *Streptomyces rochei*, and *Streptomyces* sp. [4,32].

#### 3.1.2. Utilization of Paraloids as Sole Carbon Sources

Among the recovered microorganisms, six isolates (two bacteria, two fungi, and two actinomycetes) were identified as strong acid producers and were therefore selected for further investigation. The ability of these isolates to utilize the synthetic acrylic resins Paraloid B-72, Paraloid B-66, and Paraloid B-44 as sole carbon sources was evaluated.

The results indicated that all six isolates were able to grow on the tested polymers, although with varying degrees of growth. The actinomycete isolates A1 and A3 and the fungal isolates F2 and F4 exhibited strong growth on Paraloid B-72 and Paraloid B-44, suggesting a higher capacity to utilize these polymers, illustrated in Table 1 and Table 2, respectively.

As illustrated in Table 3, the bacterial isolates B3 and B5 showed different growth responses. Isolate B5 demonstrated strong growth on Paraloid B-72 and Paraloid B-44, whereas B3 exhibited only moderate growth on the same polymers.

In contrast, Paraloid B-66 supported weaker growth for most isolates. Both bacterial and actinomycete isolates showed weak growth, while the fungal isolates exhibited moderate growth on this polymer. The observed differences in microbial growth among the tested polymers may be attributed to variations in their chemical composition and molecular structure [22,23,24,25]. Paraloid B-72 is a copolymer of ethyl methacrylate and methyl acrylate, while Paraloid B-66 is composed mainly of isobutyl methacrylate, and Paraloid B-44 is a methyl methacrylate copolymer [27,33,34]. Acrylic polymers contain ester bonds within their molecular backbone, which may become susceptible to enzymatic attack by microorganisms capable of producing esterases and other hydrolytic enzymes [35,36,37]. These enzymes can catalyze the cleavage of ester linkages, leading to depolymerization and the release of smaller organic compounds that can subsequently be utilized as carbon and energy sources by microbial cells [37,59,60,61].

The stronger growth observed on Paraloid B-72 and B-44 compared with Paraloid B-66 may be related to differences in polymer chain structure, side groups, and physicochemical properties such as molecular weight, cross-linking density, and surface hydrophobicity [22,23,24,25]. Polymers with relatively lower structural complexity or higher accessibility of ester groups may be more vulnerable to microbial colonization and enzymatic degradation [35,36,37]. In contrast, the weaker growth recorded on Paraloid B-66 may indicate a comparatively higher resistance to microbial utilization, attributable to three complementary mechanisms. First, steric hindrance—the bulky isobutyl side groups physically limit microbial esterase access to the ester carbonyl group [62]. Second, higher surface hydrophobicity the isobutyl substituents restrict the water film formation essential for microbial colonization and enzymatic activity [22,23,24,25]. Third, reduced ester bond accessibility may play a key role in limiting polymer susceptibility to degradation. Structural features of acrylic resins, particularly the arrangement and accessibility of ester linkages, can influence their resistance to hydrolytic and microbial attack. Polymers with less accessible ester groups are generally less prone to enzymatic cleavage, which may contribute to differences in degradation behavior among the tested materials [22,23,24,25,62].

The ability of bacteria, fungi, and actinomycetes to utilize these materials highlights the importance of evaluating the long-term biological stability of conservation polymers, particularly when applied to stone substrates that naturally harbor diverse microbial communities capable of contributing to biodeterioration processes.

### 3.2. Identification of Bacterial, Actinomycetes, and Fungal Isolates

Phylogenetic analysis based on the obtained sequence data revealed that the two bacterial isolates showed a high similarity with members of the genus Bacillus. Accordingly, isolate B3 was identified as *Bacillus cereus* B3 (GenBank accession no. MH923513), while isolate B5 was identified as *Bacillus subtilis* B5 (GenBank accession no. MH923514).

**Figure 5:**
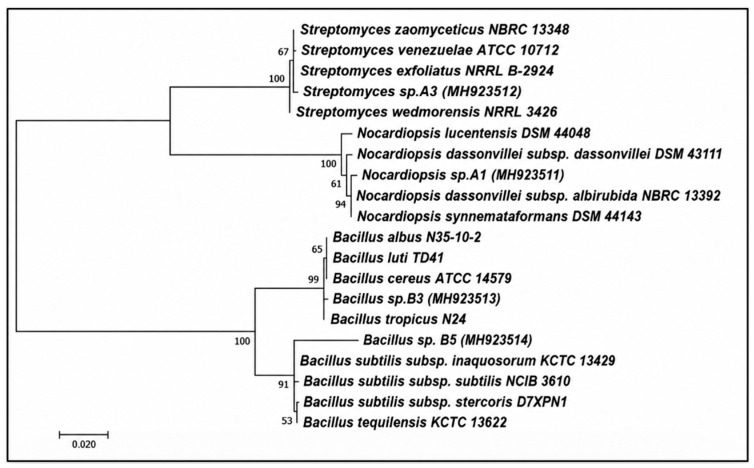
Phylogenetic relationships of the selected bacterial and actinomycete isolates based on 16S rRNA gene sequences. The tree was constructed using the neighbor-joining method, showing the relationship between the studied isolates and their closest reference strains retrieved from GenBank. Bootstrap values (%) based on 1000 replicates are indicated at branch nodes. The scale bar represents the number of nucleotide substitutions per site.

Similarly, the actinomycete isolates A1 and A3 were identified based on phylogenetic analysis as *Nocardiopsis dassonvillei* A1 (GenBank accession no. MH923511) and *Streptomyces exfoliatus* A3 (GenBank accession no. MH923512), respectively. The phylogenetic relationships of the identified bacterial and actinomycete isolates are illustrated in Figure 5.

Fungal isolates were identified based on their macroscopic and microscopic morphological characteristics. Colony morphology, including color, texture, growth pattern, and pigmentation, was first examined on culture media. Microscopic identification was subsequently performed using a light microscope, where fungal structures such as conidiophores, vesicles, phialides, and conidia were observed and compared with standard taxonomic keys. Based on these morphological characteristics, isolate F2 was identified as *Aspergillus flavus*, while isolate F5 was identified as *Penicillium crustosum*.

### 3.3. Weight Loss Measurements Before and After Consolidation

Weight loss measurements were illustrated in Table 4, these measurements revealed that limestone cubes inoculated with mixed microbial cultures exhibited higher weight reduction than those inoculated with single isolates, as presented in Table 1.

Before consolidation, the greatest weight loss was observed in cubes inoculated with the mixed bacterial population (*Bacillus* sp. B3 and *Bacillus* sp. B5), indicating their pronounced ability to induce stone deterioration.

After consolidation, the highest weight loss was recorded in cubes inoculated with the mixed fungal isolates (*Aspergillus flavus* and *Penicillium crustosum*), suggesting a strong capability of fungal communities to colonize and degrade polymer-treated stone surfaces.

Regarding the tested consolidants, the highest weight loss after treatment was observed in cubes consolidated with Paraloid B-72, followed by Paraloid B-44, whereas the lowest weight loss was associated with Paraloid B-66, indicating a comparatively higher resistance of this polymer to microbial deterioration.

Table 4 show clear differences in microbial growth patterns were observed among the tested polymers, with Paraloid B-72 and B-44 generally supporting stronger microbial activity compared with B-66, indicating variations in susceptibility to biodegradation.

The data presented in this table allow for qualitative comparison of the relative growth behavior and biodegradation potential of different microbial groups across the tested Paraloid resins. The observed trends highlight differences in microbial activity depending on both polymer type and microbial composition.

### 3.4. Visual and Stereo Microscope Observation

#### 3.4.1. Before Consolidation

**Figure 6:**
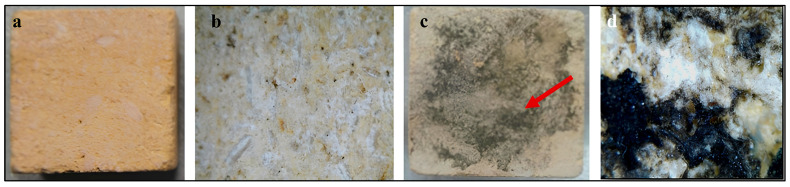
Visual and stereomicroscopic examination of limestone samples before consolidation. (**a**) Untreated control limestone under visual observation showing a homogeneous surface. (**b**) Stereomicroscopic image of the control sample. (**c**) Limestone sample inoculated with mixed actinomycete cultures showing visible surface discoloration (the arrow indicates darkened areas associated with microbial growth). (**d**) Stereomicroscopic image of the inoculated sample illustrating microbial colonization and surface alteration.

Figure 6 shows visual and stereomicroscopic examination of limestone cubes before and after microbial inoculation. (Figure 6a) Untreated control limestone cube photographed under normal visual observation showing an intact and homogeneous surface. (Figure 6b) Stereomicroscopic image of the control sample revealing the natural microstructure of the limestone without any signs of biological colonization. (Figure 6c) Limestone cube inoculated with mixed actinomycete cultures showing visible surface discoloration and the beginning of microbial colonization. (Figure 6d) Stereomicroscopic image of the inoculated sample illustrating dense microbial growth and dark brown to black surface pigmentation (indicated by the arrow), which represents microbial colonization and early biodeterioration features on the limestone surface.

#### 3.4.2. After Consolidation

**Figure 7:**
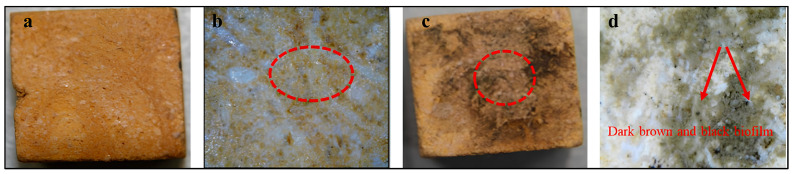
Visual and stereomicroscopic examination of limestone samples after consolidation with acrylic polymers. (**a**) Consolidated control limestone under visual observation showing a relatively uniform surface. (**b**) Stereomicroscopic image of the consolidated control sample illustrating the penetration of the polymer into stone pores and the formation of a thin binding film (dashed circle). (**c**) Consolidated limestone after microbial inoculation, red dashed circle showing localized discoloration and surface staining. (**d**) Stereomicroscopic image of the inoculated consolidated sample revealing dense microbial colonization and biofilm formation (arrows), associated with deterioration of the polymer-treated surface.

Figure 7 shows visual and stereomicroscopic examination of consolidated limestone cubes before and after microbial colonization. In Figure 7a, consolidated limestone cube photographed under normal visual observation showing a relatively homogeneous surface after polymer application. (Figure 7b) Stereomicroscopic image of the consolidated control sample illustrating the penetration of the consolidant into the stone pores and the formation of a thin polymeric film binding the stone grains (dashed circle). (Figure 7c) Consolidated limestone cube after microbial inoculation showing localized surface discoloration and brown staining resulting from microbial activity. (Figure 7d) Stereomicroscopic image of the infected consolidated sample revealing dense microbial colonization and the formation of dark brown to black biofilm (indicated by arrows), associated with biodeterioration processes affecting the polymer-treated limestone surface.

### 3.5. Compressive Strength Test

**Figure 8:**
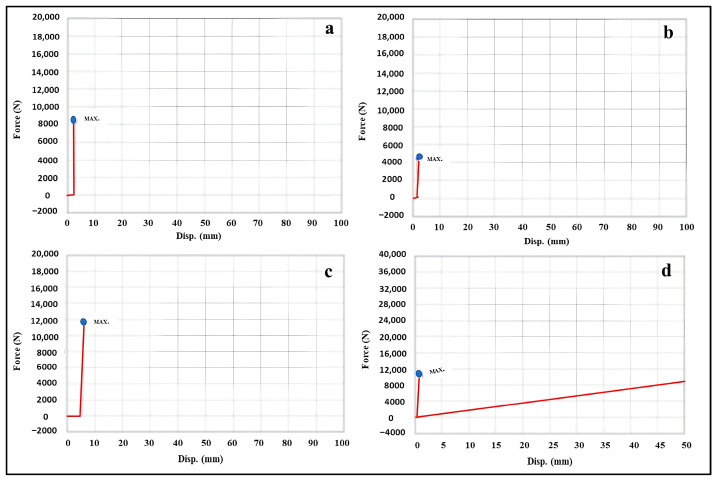
Force-displacement curves obtained from compressive strength testing of limestone samples under different treatment conditions. The blue dot indicates the maximum force value, while the red line represents the force–displacement behavior during the test. (**a**) Untreated control limestone before consolidation, showing the inherent mechanical behavior of the stone. (**b**) Limestone sample inoculated with mixed bacterial isolates before consolidation, illustrating the reduction in mechanical strength due to microbial deterioration. (**c**) Consolidated control limestone, demonstrating the improvement in compressive strength following polymer application. (**d**) Consolidated limestone after microbial inoculation, showing altered mechanical behavior and reduced strength as a result of biodeterioration. Variations in peak force and displacement reflect changes in internal cohesion and structural integrity of the limestone.

As illustrated in Table 5 and Figure 8a–d, the compressive strength test was carried out to evaluate the mechanical integrity of limestone cubes before and after consolidation and microbial inoculation. The results revealed that the control limestone cube exhibited the highest maximum force before consolidation (8322.70 N), indicating the natural mechanical strength of the untreated limestone.

Microbial inoculation resulted in a noticeable reduction in compressive strength. Among the inoculated samples, the limestone cube inoculated with the mixed bacterial population (*Bacillus* sp. B3 and *Bacillus* sp. B5) exhibited the highest maximum force before consolidation (4447.78 N), whereas the lowest value was recorded for the actinomycete-inoculated sample (2616.13 N), suggesting that microbial activity contributed to the weakening of the stone matrix.

After consolidation, a significant increase in compressive strength was observed in the control sample, reaching 23,040 N, which indicates the effectiveness of the applied consolidant in improving the mechanical cohesion of the limestone. Similarly, an increase in maximum force was recorded for some inoculated samples, particularly the bacterial and fungal mixed populations (10,819.5 N and 14,043.5 N, respectively), suggesting that the consolidant partially restored the mechanical stability of the deteriorated stone. Regarding displacement values, the highest displacement was recorded in cubes inoculated with mixed actinomycete populations after consolidation, indicating greater deformation of the stone before failure. This behavior may be attributed to microbial-induced weakening of the stone structure, which allows greater deformation under compressive stress [63,64].

### 3.6. Scanning Electron Microscope (SEM)

**Figure 9:**
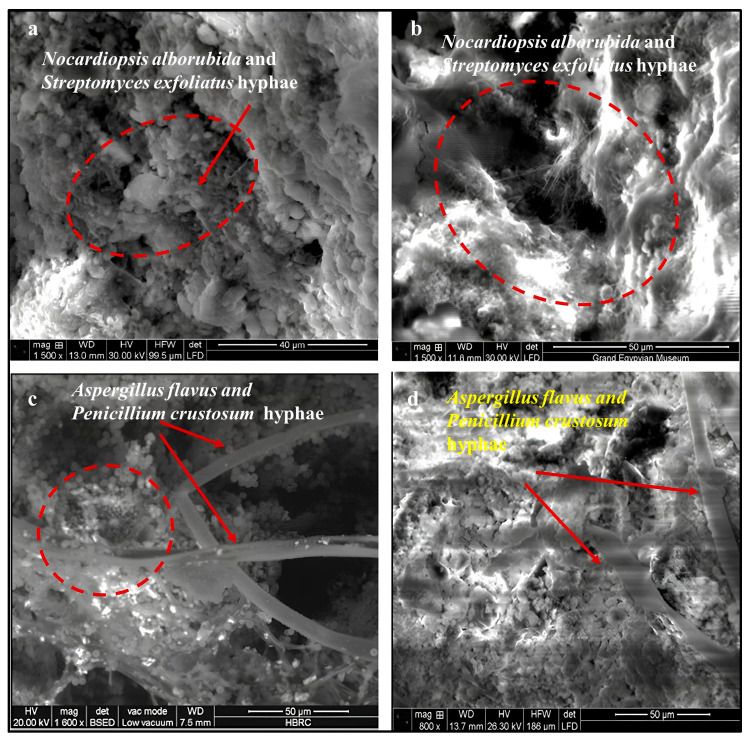
Scanning electron microscope (SEM) photomicrographs illustrating the biodeterioration of limestone surfaces before and after consolidation with acrylic polymers. (**a**) Limestone surface before consolidation inoculated with *Nocardiopsis alborubida* and *Streptomyces exfoliatus* (1500×, 30 kV, 40 µm scale bar); the red circle highlighted spherical fine actinomycete filaments, while the calcite matrix largely retains its original crystalline structure (**b**) Limestone surface after consolidation with acrylic polymer, inoculated with *Nocardiopsis alborubida* and *Streptomyces exfoliatus* (1500×, 30 kV, 50 µm scale bar); (**c**) Limestone before consolidation inoculated with *Aspergillus flavus* and *Penicillium crustosum* (1600×, 20 kV, 50 µm scale bar, BSED mode); (**d**) Limestone after consolidation inoculated with *Aspergillus flavus* and *Penicillium crustosum* (800×, 26.30 kV, 50 µm scale bar). Red arrows points to dense fungal hyphae penetrated the disrupted acrylic film, indicating polymer degradation caused by fungal esterase activity.

The SEM micrograph in Figure 9a shows the inoculated limestone cube with actinomycetes (*N. alborubida* + *S. exfoliatus*) (and) before consolidation, displaying a moderately cohesive granular surface with early microbial colonization mainly in intergranular spaces. Fine actinomycete filaments are present but remain superficial, while the calcite matrix largely retains its original crystalline structure, indicating early-stage bio-geophysical weathering without significant structural damage [65].

In Figure 9b, after consolidation cube was inoculated with actinomycetes (*N. alborubida* + *S. exfoliatus*). The interaction between the actinomycetes community and the treated substrate is markedly more destructive. The dashed circle highlights a large irregular void—likely the result of localized polymer degradation and dissolution of the underlying calcite—surrounded by a heterogeneous surface of collapsed and altered material. The roughened, cauliflower-like surface texture suggests biofilm-driven chemical weathering, where actinomycetes metabolites (organic acids, enzymes) attacked both the polymeric consolidant and the calcareous substrate simultaneously [31,66].

In Figure 9c, the (BSED mode) micrograph shows the inoculated sample with *Aspergillus flavus* and *Penicillium crustosum* before consolidation. The image reveals thick, ribbon-like hyphae bridging and separating calcite grains [55]. Arrows highlight zones of mechanical intrusion where hyphal growth exerts wedging pressure along crystal boundaries. The dashed area marks localized grain disintegration and inter-crystalline loosening, attributed to combined mechanical stress and acid-mediated microenvironments generated by fungal activity [67].

In Figure 9d, SEM micrographs shows the consolidated sample inoculated with *Aspergillus flavus* and *Penicillium crustosum*, exhibiting the most advanced deterioration. Dense, branching hyphae penetrate and traverse the disrupted acrylic layer, indicating degradation of the polymer film. The acrylic film appears disrupted and fragmented, consistent with fungal esterase activity hydrolyzing the ester bonds within the Paraloid backbone. Crucially, the hyphae appear to have used the polymer matrix as a carbon and energy source, proliferating vigorously and extending into the underlying limestone [12,13,14,15,16,17].

### 3.7. X-Ray Diffraction Measurements

**Figure 10:**
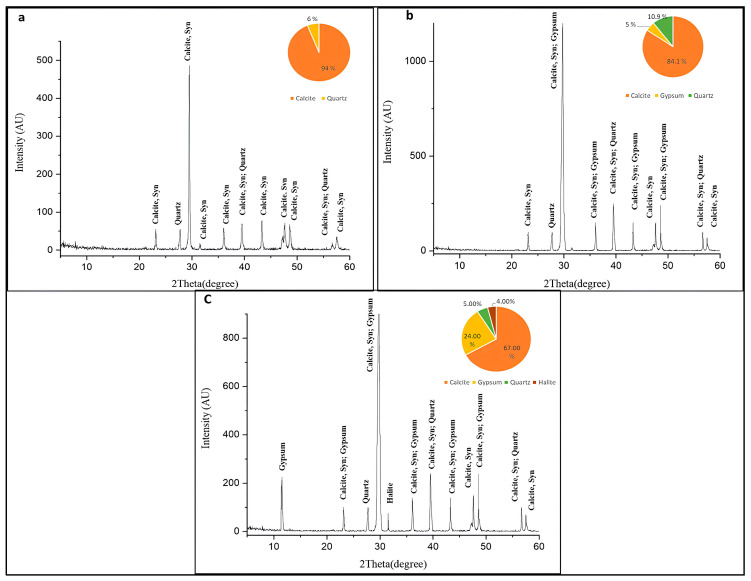
XRD diffractograms (Cu Kα radiation) of control limestone samples before inoculation (**a**), inculcated with *Nocardiopsis alborubida* and *Streptomyces exfoliatus* before consolidation (**b**), and after consolidation with acrylic polymer (**c**), with inset pie charts showing the relative mineral phase proportions identified using ICDD reference cards. Dominant phases are labeled at their corresponding 2θ reflection positions.

The XRD results in Figure 10a indicate that the control stone is predominantly composed of calcite (94%), with a minor quartz content (6%), suggesting a calcitic limestone of relatively high purity. The identification of calcite corresponds to the stable polymorph of calcium carbonate, while further microstructural characterization would be required to distinguish between micritic and sparitic textures. XRD pattern of inoculated sample before consolidation shown in Figure 10b. The diffractogram is dominated by sharp, high-intensity reflections characteristic of well-crystallized calcite (CaCO_3_), with the strongest peak appearing at approximately 2θ = 29.4°—the diagnostic (104) reflection of calcite. Additional calcite reflections are visible at 2θ ≈ 23°, 36°, 39°, 43°, 47–48°, 57°, and 60–61°, confirming calcite as the overwhelmingly predominant mineral phase. A secondary quartz (SiO_2_) reflection is clearly resolved at 2θ ≈ 26.6°. Importantly, low-intensity gypsum (CaSO_4_·2H_2_O) peaks are identifiable co-occurring with several calcite reflections in the 29–48° range, though at comparatively subdued intensity. The inset pie chart quantifies the phase composition as calcite 84.2%, quartz 10.9%, and gypsum 5%, confirming that microbial sulfur metabolism by *N. alborubida* and *S. exfoliatus* has already initiated sulfation of the calcite surface, converting a small but measurable fraction of the stone to gypsum prior to polymer application [4,68].

In Figure 10c, the inoculated post-consolidation diffractogram reveals a profoundly altered mineralogical profile. The most striking feature is the emergence of two strong, well-resolved gypsum peaks at 2θ ≈ 11.6° and 20.7°—characteristic of the (020) and (021) reflections of gypsum—which are entirely absent in pattern (b). These prominent low-angle peaks signal a substantial increase in gypsum crystallinity and abundance. The calcite dominant peak at 2θ ≈ 29.4° remains the strongest reflection but shows a marked reduction in relative intensity, and the peak labels now frequently read as “Calcite; Gypsum” rather than pure calcite, indicating peak overlap due to increased gypsum content [4,68,69]. A new reflection attributable to halite (NaCl) appears at 2θ ≈ 31.7°, suggesting salt crystallization as an additional deterioration product [68]. The inset pie chart now records calcite at 67%, gypsum at 24%, quartz at 5%, and an additional minor phase (likely halite) at 4%—representing a nearly five-fold increase in gypsum content relative to the pre-consolidation sample.

The dramatic increase in gypsum from 5% to 24% after consolidation directly reflects the enhanced metabolic activity of the actinomycetes on the polymer-treated substrate [68]. The acrylic Paraloid resin, being an organic carbon source, fueled more vigorous microbial growth, which in turn elevated the production of sulfuric and organic acids [12,13,14,15,16,17]. These acids attacked the calcite substrate via the reaction CaCO_3_ + H_2_SO_4_ → CaSO_4_·2H_2_O, progressively converting the structural calcite into gypsum—a conversion that is not only chemically irreversible under ambient conditions but is physically destructive, as the volumetric expansion associated with gypsum crystallization generates internal stresses that accelerate cracking, spalling, and surface loss [4,68]. The appearance of halite in pattern (c) further suggests that the microbial activity promoted ion mobilization and evaporative salt deposition at the stone surface [4]. Together, these XRD data provide robust mineralogical evidence that consolidation with Paraloid resins, in the presence of bio-deteriorating actinomycetes, significantly accelerates the chemical weathering of limestone rather than protecting it.

### 3.8. FT-IR Spectroscopy

**Figure 11:**
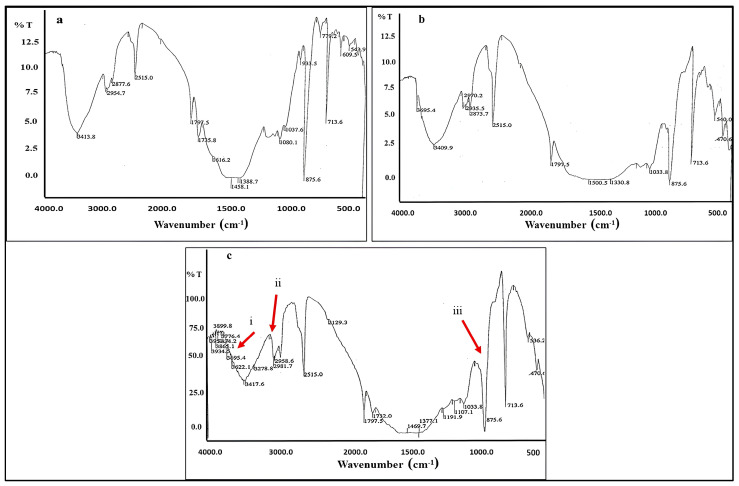
FTIR transmittance spectra (4000–500 cm^−1^) of limestone samples under three experimental conditions. (**a**) Control limestone sample (uninoculated, unconsolidated), showing the characteristic absorption profile of a calcite substrate. (**b**) Inoculated limestone sample (without consolidation) exposed to *Nocardiopsis alborubida* and *Streptomyces exfoliatus*, revealing spectral modifications attributable to microbial metabolic activity and incipient biodeterioration. (**c**) Consolidated limestone sample (Paraloid acrylic resin) before microbial inoculation; red arrows indicate the diagnostic absorption bands of the Paraloid polymer matrix—(i) C–H stretching region (~2900–3400 cm^−1^), (ii) carbonyl C=O ester stretching (~1730 cm^−1^ region, broad), and (iii) C–O–C ester linkage and aliphatic C–H deformation bands (~1033–1191 cm^−1^)—all of which show evidence of structural alteration following microbial colonization.

FTIR spectrum in Figure 11a, show the baseline mineralogical fingerprint of the control limestone substrate. The key absorptions are: ~3413 cm^−1^: Broad O–H stretching band associated with adsorbed moisture, ~2954 and 2877 cm^−1^: Weak C–H stretching bands, likely attributable to trace organic matter naturally present in the limestone matrix [68,69].

~2515 cm^−1^: A characteristic combination overtone band of calcite (CO_3_^2−^), diagnostic of CaCO_3_ and consistent with the dominant calcite phase identified in the control sample. ~1797 and 1735 cm^−1^: Weak absorptions in the carbonyl region; the 1797 cm^−1^ band is a known overtone of the calcite carbonate asymmetric stretching mode [69]. ~1616 cm^−1^: O–H bending, ~1458 and 1388 cm^−1^: Strong asymmetric and symmetric C–O stretching vibrations of the CO_3_^2−^ group in calcite—among the most diagnostic bands for carbonate minerals [68,69].

~875 cm^−1^: Out-of-plane bending (ν_2_) of CO_3_^2−^ in calcite—a sharp, diagnostic peak. ~713 cm^−1^: In-plane bending (ν_4_) of CO_3_^2−^ in calcite [69]. Overall, the control spectrum is dominated by calcite signatures, providing a clean reference baseline against which microbial and polymer-induced changes can be assessed.

Figure 11b shows the inoculated limestone before consolidation, the spectrum concludes that, ~3595 and 3409 cm^−1^: The O–H stretching region is broader and shifted relative to the control, with the emergence of a shoulder at ~3595 cm^−1^—a characteristic doublet of crystalline gypsum (corresponding to the two non-equivalent water molecules in its structure) [4,68]. This intensification directly reflects the presence of gypsum as calcite undergoes sulfation driven by microbial sulfuric and organic acid production [4,68,69].

~2970, 2935, and 2873 cm^−1^: The C–H stretching region shows notably enhanced absorption compared to the control. These bands, attributable to –CH_3_ and –CH_2_ stretching vibrations, indicate the accumulation of microbial biomass, and metabolic organic by-products on and within the stone surface [1,4,68].

~2515 cm^−1^: The calcite overtone band is retained, confirming residual calcite, but its relative intensity appears reduced against the background, consistent with partial calcite dissolution [68]. ~1797 cm^−1^: Maintained, confirming persisting calcite. ~1500 and 1330 cm^−1^: Broadening and slight shifts in the carbonate stretching region compared to the sharp 1458/1388 cm^−1^ doublet of the control. This broadening suggests partial distortion of the calcite crystal lattice or overlap with C–O contributions from microbial organic acids (oxalic, citric, gluconic) adsorbed on the stone surface [1,4,69]. ~1033 cm^−1^: The SO_4_^2−^ stretching band of gypsum is illustrated in contrast with the control, corroborating the XRD-measured presence of gypsum content. ~875 and 713 cm^−1^: Calcite bending bands are retained but show slight reduction in transmittance depth relative to the control, consistent with partial carbonate degradation [69]. ~540 and 470 cm^−1^: SO_4_^2−^ bending modes of gypsum appears clearly [1,4,69].

The spectrum of panel (b) therefore documents the chemical consequences of microbial biodeterioration on bare limestone all consistent with the weight loss and compressive strength data.

In Figure 11c, the sample was consolidated and inoculated (Paraloid + microorganisms). The three red arrows identify the key diagnostic contributions of the Paraloid acrylic resin and their post-inoculation modifications: Arrow (i)—~2900–3400 cm^−1^ region (C–H stretching of Paraloid): The cluster of peaks at 3899, 3776, 3456, 3422, 3278, 3222, and 2981–2934 cm^−1^ collectively represent the aliphatic C–H stretching vibrations of the acrylic polymer backbone, specifically the ethyl methacrylate and methyl acrylate ester side chains characteristic of Paraloid B-72 [70]. The exceptional complexity and breadth of this region, compared to the relatively simple O–H/C–H envelope in panels (a) and (b), unambiguously confirms the presence of the acrylic consolidant. Crucially, the intensity and profile of these bands suggests partial degradation of the polymer chains, consistent with microbial esterase-mediated depolymerization of the acrylic backbone [70,71].

Arrow (ii)—~2258–2515 cm^−1^ region (polymer-related and calcite overtone): This region in the consolidated sample shows a distinctive absorption near 2258 cm^−1^ that is absent in panels (a) and (b). In acrylic polymers, absorptions in this range can be associated with overtone or combination bands of ester carbonyl groups. The persistence and shape of the 2515 cm^−1^ calcite overtone confirms that the underlying limestone substrate is still present, while the additional absorption near 2258 cm^−1^ is polymer-specific. Its presence in the inoculated consolidated sample warrants attention as it may reflect structural rearrangement or oxidative modification of the polymer under microbial attack [72].

Arrow (iii)—~1033–1191 cm^−1^ region (C–O–C ester stretching of Paraloid): The strong, complex absorption envelope spanning 1033–1191 cm^−1^, with resolved peaks at 1191, 1107, 1033 cm^−1^, is characteristic of the C–O–C asymmetric and symmetric stretching vibrations of the ester linkages within the acrylic polymer backbone [70,71]. In pristine Paraloid, these bands are sharp and well-defined. In the inoculated consolidated sample, the relative intensities and positions of these bands are altered notably, the broadening of the 1033 cm^−1^ band and the emergence of the 1107 cm^−1^ shoulder suggest partial hydrolysis of the ester bonds by microbial esterases. This finding provides direct spectroscopic evidence for enzymatic degradation of the consolidant [70,71,72].

Additionally, the 1797 cm^−1^ calcite overtone is retained, and the 875 and 713 cm^−1^ carbonate bending peaks remain present, indicating that the limestone substrate beneath the polymer layer has not been completely dissolved, though its relative proportion has diminished.

## 4. Discussion

The present study demonstrates that microbial communities isolated from archeological limestone are capable of actively contributing to the degradation of both acrylic consolidants and the underlying stone substrate. This deterioration is primarily driven by acid production, enzymatic activity, and biofilm formation, all of which are well-recognized mechanisms in the biodeterioration of calcareous materials [73,74].

The higher microbial counts obtained using the grinding method compared with swabbing and adhesive tape techniques can be attributed to its ability to recover microorganisms not only from the surface but also from deeper pores and microfractures within the limestone matrix [75,76]. This observation is consistent with previous studies, such as Abdulla et al. 2008 [32], who reported similar findings when investigating biodeterioration of limestone in archeological contexts. While non-destructive methods remain valuable for conservation purposes, the grinding approach appears to provide a more representative assessment of total microbial load [75,76,77].

The ability of the selected isolates to grow on Paraloid resins as sole carbon sources confirms that these commonly used conservation materials are susceptible to microbial colonization and biodegradation [67,78,79,80,81]. This finding is in agreement with previous reports indicating that acrylic resins, including Paraloid B-72, are not biologically inert and may support microbial growth under suitable environmental conditions [31,82,83,84]. Studies by Pinna and Salvadori (1999) [85], Kigawa et al. (2005) [86], and Cappitelli et al. (2021) [83] have similarly demonstrated the colonization and degradation of synthetic polymers by fungi and other microorganisms associated with cultural heritage materials.

The observed differences in microbial growth among the tested polymers highlight the influence of polymer composition and structure on biodegradability [87]. Stronger growth on Paraloid B-72 and B-44, compared with B-66, may be attributed to differences in side-chain structure, ester bond accessibility, and surface properties such as hydrophobicity [83,88]. These structural factors likely influence enzymatic accessibility and microbial adhesion, thereby affecting the susceptibility of each polymer to biodegradation [89]. Importantly, this study provides a direct comparative evaluation of three widely used Paraloid resins under identical conditions, offering insights into their relative resistance to microbial attack.

Weight loss measurements further supported the occurrence of biodegradation, with the highest values recorded in samples treated with Paraloid B-72 and exposed to mixed microbial cultures [89]. The enhanced deterioration observed in mixed cultures can be explained by synergistic interactions among microorganisms, where combined metabolic activities lead to increased production of organic acids and degradative enzymes [90]. This finding emphasizes the importance of considering microbial consortia rather than single isolates when evaluating biodeterioration processes under realistic conditions [90].

The reduction in compressive strength following microbial inoculation indicates a loss of internal cohesion within the limestone matrix [91,92]. This weakening can be attributed to microbial penetration, dissolution of calcite, and the production of extracellular polymeric substances (EPS), which contribute to mechanical stress and the development of microcracks. Similar observations have been reported in previous studies investigating the impact of microbial activity on carbonate stones [93,94,95].

A limitation of this part is the absence of replicate measurements for compressive strength tests, which prevents the application of statistical analysis such as standard deviation and significance testing. As a result, the reported values should be interpreted as indicative trends rather than statistically confirmed differences. Future studies should incorporate multiple replicates to enable robust statistical evaluation of treatment effects.

SEM observations provided direct visual evidence of biodeterioration, revealing disintegration between calcite grains, cracking of the polymer matrix, and extensive microbial colonization. These microstructural alterations are consistent with combined biogeochemical and bio-geophysical processes, including acid-mediated mineral dissolution and physical disruption associated with microbial growth and EPS dynamics [96,97,98].

XRD analysis demonstrated a significant increase in gypsum formation following microbial activity, indicating enhanced mineral transformation of calcite. This suggests that the presence of polymeric consolidants may, under certain conditions, promote rather than inhibit microbial deterioration by providing an additional carbon source that stimulates microbial metabolism [95]. These findings are consistent with previous studies reporting mineralogical changes in consolidated limestone subjected to environmental and biological stress [99].

FTIR spectra revealed noticeable alterations in the characteristic absorption bands of the acrylic polymers following microbial exposure [70,71,72,99]. In particular, changes in the intensity and shape of the ester carbonyl band (~1730 cm^−1^), along with the appearance and/or enhancement of bands in the hydroxyl (~3400 cm^−1^) and C–O (~1000–1200 cm^−1^) regions, suggest partial hydrolysis of ester bonds within the polymer structure [70,71]. These spectral modifications are consistent with microbial enzymatic activity leading to depolymerization of acrylic resins [20,21]. However, it should be noted that the present analysis is primarily qualitative. A detailed quantitative evaluation of FTIR peak ratios would be required to confirm the extent of ester bond cleavage and to accurately assess the degree of polymer degradation.

Nevertheless, the agreement between independent analytical techniques supports the proposed degradation mechanism at a qualitative level.

The combined results of microbial growth, weight loss, and FTIR analysis suggest a consistent pattern of polymer degradation, the present study does not provide a quantitative correlation between microbial activity and the rate of chemical transformation. Establishing such a relationship would require time-resolved measurements and kinetic analysis, which were beyond the scope of this work.

Although microbial degradation of synthetic polymers has been reported in previous studies, the present work extends this knowledge by providing a comparative and integrated assessment under conservation-relevant conditions. In particular, linking polymer biodegradation to measurable mechanical and mineralogical deterioration of limestone offers a more comprehensive understanding of the risks associated with the use of acrylic consolidants.

It should also be noted that the present study was not designed as a full factorial experiment, and therefore interaction effects between variables were not statistically evaluated. While this approach allows for targeted comparison of specific conditions, future studies employing design of experiments (DOE) methodologies would provide a more comprehensive assessment of factor interactions.

However, a limitation of the present study is the absence of an uninoculated polymer-treated control sample. The inclusion of such a control would allow a clearer distinction between abiotic aging processes (e.g., hydrolysis or environmental degradation of the polymer) and true microbial biodegradation. Although the observed chemical and structural changes are strongly associated with microbial activity, it cannot be entirely excluded that some alterations may result from non-biological factors. Therefore, the results should be interpreted with caution, and future studies should incorporate polymer-only controls to better differentiate between abiotic and biotic degradation mechanisms.

Another limitation is the use of a relatively small number of selected microbial isolates, primarily chosen based on their strong acid-producing capacity. While this approach allows for the evaluation of a worst-case scenario and highlights the maximum potential for biodeterioration, it does not fully represent the diversity and complexity of natural microbial communities present on archeological limestone. In natural environments, microbial populations include a wide range of organisms with varying metabolic capabilities, including weak and non-acid-producing species. Consequently, the observed deterioration effects may represent an intensified scenario compared with real field conditions.

Overall, the findings of this study indicate that commonly used acrylic consolidants, particularly Paraloid B-72, may not provide sufficient long-term protection against biodeterioration under microbiologically active conditions. Instead, they may contribute to the deterioration process by supporting microbial growth and promoting chemical alteration of the stone substrate [100]. These results highlight the need for the development of more resistant conservation materials and for evaluating consolidants under realistic environmental and microbiological conditions.

## 5. Conclusions

The present study provides clear evidence that synthetic polymers commonly used as stone consolidants are not biologically inert, but can actively participate in biodeterioration processes under microbial colonization. The recovered microbial community, including bacteria, fungi, and actinomycetes, demonstrated varying abilities to produce acids and utilize synthetic polymers as sole carbon sources, confirming their metabolic adaptability in nutrient-limited environments such as stone substrates.

The predominance of acid-producing isolates, particularly among fungal species, highlights the critical role of organic acid production in stone deterioration. Organic acids are known to solubilize calcium carbonate, leading to mineral dissolution and structural weakening of limestone. This was clearly reflected in the weight loss measurements, where inoculated samples exhibited significantly higher mass reduction compared to controls, especially under mixed microbial cultures. The enhanced deterioration observed in mixed populations can be attributed to three synergistic mechanisms: metabolic cross-feeding, whereby fungal esterases hydrolyze Paraloid ester bonds (Figure 10c), releasing monomers that sustain secondary community members beyond what single isolates achieve; pH-mediated cooperative attack, whereby acid-producing organisms lower local pH, accelerating ester bond hydrolysis and CaCO_3_ dissolution, as evidenced by the five-fold gypsum increase (5% → 24%, Figure 9) and void formation (Figure 8b,d); and biofilm EPS matrix effects, whereby extracellular polymeric substances retain moisture and concentrate hydrolytic enzymes at the polymer–stone interface, sustaining active deterioration throughout the 4-week incubation period (Figure 6d and Figure 7d).

The ability of the selected isolates to grow on Paraloid B-72, B-66, and B-44 confirms that these acrylic polymers can serve as potential carbon and energy sources. The differential growth observed among the tested polymers suggests that their susceptibility to microbial attack is strongly influenced by their chemical composition and molecular structure. The relatively higher biodegradability of Paraloid B-72 and B-44 compared to B-66 may be related to the accessibility of ester bonds within their polymer backbone, which are susceptible to enzymatic hydrolysis by microbial esterases. In contrast, the lower susceptibility of B-66 may be attributed to steric hindrance and its more hydrophobic structure, which can limit microbial enzymatic access.

The mechanical properties of limestone were significantly influenced by both microbial activity and consolidation treatment. Prior to consolidation, microbial colonization led to a marked reduction in compressive strength, confirming the weakening effect of biodeterioration. Although consolidation improved the mechanical strength of the limestone, particularly in control samples, the persistence of strength reduction in inoculated samples indicates that microbial activity can compromise the effectiveness of consolidants over time. This suggests that consolidation alone may not be sufficient to ensure long-term preservation under biologically active conditions.

Microscopic observations using stereomicroscopy and SEM further supported these findings by revealing clear evidence of microbial colonization, including biofilm formation, surface discoloration, and microstructural alterations. The development of dark brown to black biofilms on consolidated samples indicates that polymer-treated surfaces may provide favorable conditions for microbial attachment and growth. Biofilms not only protect microorganisms from environmental stress but also enhance their ability to degrade substrates through localized concentration of enzymes and metabolites.

FTIR analysis provided additional insights into the chemical alterations associated with microbial activity. The identification of characteristic polymer bands confirmed the presence of the consolidants within the stone matrix, while the appearance of additional absorption bands and changes in peak intensity suggest possible chemical modifications of the polymer structure. These changes may be attributed to microbial-induced processes such as oxidation, hydrolysis of ester bonds, and chain scission, which ultimately lead to polymer degradation. The coexistence of calcite-related bands alongside polymer signals further confirms that degradation processes affect both the stone substrate and the applied consolidants.

Overall, the integration of microbiological, chemical, and mechanical analyses demonstrates that synthetic polymers, despite their widespread use in conservation, may contribute to biodeterioration when exposed to active microbial communities. These findings emphasize the importance of evaluating the long-term biological stability of conservation materials and highlight the need for developing more resistant or bio-inhibitive consolidants to ensure the durability of treated stone artifacts.

## Figures and Tables

**Table 1:** Utilization of Paraloids as sole carbon source by actinomycetes isolates (n = 3). (−) negative results, (+) weak growth, (++) moderate growth, (+++) strong growth.

	Actinomycete Isolates	Paraloid B-72	Paraloid B-66	Paraloid B-44
A_1_	*Nocardiopsis alborubidus*	+++	+	+++
A_2_	*Nocardiodes luteus*	−	−	−
A_3_	*Sreptomyces exofoliatus*	+++	+	+++
A_4_	*Streptomyces rochei*	−	−	−
A_5_	*Streptomyces* sp.	−	+	−

**Table 2:** Utilization of Paraloids as sole carbon source by fungal isolates (n = 3). (−) negative results, (+) weak growth, (++) moderate growth, (+++) strong growth. Sp. 1 and sp. 2 are two morphologically distinct isolates of the genus Penicillium that could not be identified to species level.

	Fungal Isolates	Paraloid B-72	Paraloid B-66	Paraloid B-44
F_1_	*Aspergillus niger*	−	−	+
F_2_	*Aspergillus flavus*	+++	++	+++
F_3_	*Alternaria* sp.	−	−	−
F_4_	*Penicillium* sp. 1	+++	++	+++
F_5_	*Penicillium* sp. 2	+	−	−

**Table 3:** Utilization of Paraloids as sole carbon source by eubacterial isolates (n = 3). (−) negative results, (+) weak growth, (++) moderate growth, (+++) strong growth.

	Bacterial Isolates	Paraloid B-72	Paraloid B-66	Paraloid B-44
B_1_		−	−	−
B_2_	*Bacillus cereus*	++	+	++
B_3_		−	−	−
B_4_	*Bacillus subtilis*	+++	+	+
B_5_		−	−	−

**Table 4:** Comparative evaluation of microbial growth and biodegradation potential of different microbial groups on Paraloid resins under controlled experimental conditions. Values are expressed as mean ± SD (n = 3). MA: mixed population of actinomycetes; MB: mixed population of bacteria; MF: mixed population of fungi.

	Before Consolidation		After Consolidation	
		Paraloid B-72	Paraloid B-66	Paraloid B-44
Control		0.05 ± 0.00		
Actinomycete Isolates				
*Nocardiopsis alborubidus*	0.13 ± 0.02	0.14 ± 0.02	0.07 ± 0.02	0.14 ± 0.05
*Streptomyces exofoliatus*	0.15 ± 0.03	0.16 ± 0.02	0.09 ± 0.05	0.14 ± 0.02
MA	0.28 ± 0.02	0.24 ± 0.01	0.18 ± 0.04	0.29 ± 0.04
Bacterial Isolates				
*Bacillus* sp. B3	0.11±0.01	0.18 ± 0.04	0.15 ± 0.03	0.22 ± 0.08
*Bacillus* sp. B5	0.11 ± 0.01	0.16 ± 0.06	0.09 ± 0.00	0.14 ± 0.06
MB	0.19 ± 0.04	0.28 ± 0.05	0.18 ± 0.04	0.18 ± 0.03
Fungal Isolates				
*Aspergillus flavus*	0.09 ± 0.00	0.21 ± 0.03	0.14 ± 0.01	0.24 ± 0.07
*Penicillium crustosum*	0.15 ± 0.02	0.17 ± 0.07	0.15 ± 0.01	0.24 ± 0.06
MF	0.21 ± 0.05	0.23 ± 0.08	0.15 ± 0.03	0.36 ± 0.09

**Table 5:** Compressive strength test was carried out to evaluate the mechanical integrity of limestone cubes before and after consolidation and microbial inoculation (n = 1 per group).

Name	Maximum Force (mm)	Maximum Displacement (N)
Before Consolidation		
Control	8322.70	2.11
MA	2616.13	1.26
MB	**4447.78**	**1.92**
MF	3109.78	0.86
After Consolidation		
Control	23,040.0	2.32888
MA	1677.54	**2.19**
MB	**10,819.5**	0.43
MF	14,043.5	1.18

## Data Availability

All available data including in the manuscript.

## Abbreviations

The following abbreviations are used in this manuscript:

SEM	Scanning Electron Microscopy
XRD	X-Ray Diffraction
FTIR	Fourier Transform Infra-Red
SM	Sterio Microscopy
BSED	Back-Scattered Electron Detector
EPS	Extracellular Polymeric Substances
